# Translation, Pdcd4 and eIF4A

**DOI:** 10.18632/oncoscience.192

**Published:** 2015-08-10

**Authors:** Abhiruchi Biyanee, Priyanka Singh, Karl-Heinz Klempnauer

**Affiliations:** Institute for Biochemistry, University of Muenster, Muenster, Germany

**Keywords:** Pdcd4, translation, eIF4A

**Figure 1:**
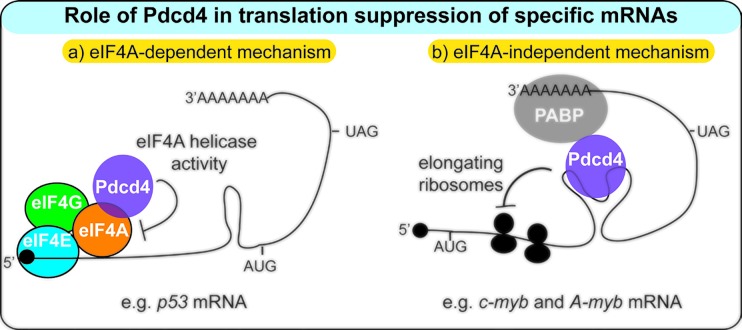
Suppression of translation initiation (a) and translation elongation (b) by Pdcd4

Pdcd4 (programmed cell death 4) has received considerable attention as a tumor suppressor protein in recent years, however, its molecular function is still poorly understood. Pdcd4 is a nuclear-cytoplasmic shuttling and RNA-binding protein, which is involved in the control of translation of specific mRNAs. Pdcd4 interacts with the eukaryotic translation initiation factor eIF4A, an RNA helicase that plays a critical role in cap-dependent translation by melting stable RNA secondary structures in the 5′-untranslated regions (UTRs) of mRNAs [1,2]. It has been shown that Pdcd4 inhibits the helicase activity of eIF4A, suggesting that it suppresses translation of mRNAs with highly structured 5′-UTRs [3]. This idea was supported by analyzing the effect of Pdcd4 on artificial RNA constructs containing stable hairpin structures in the 5′-UTR and, more recently, confirmed by demonstrating that translation of p53 mRNA (whose 5′-UTR forms very stable secondary structures) is suppressed by Pdcd4 via an eIF4A-dependent mechanism [4] (Figure 1a).

Recent findings indicate that the role of Pdcd4 in translation is more complex and involves an additional, entirely different inhibitory mechanism. It was shown that Pdcd4 suppresses the translation of the c-*myb* and A-*myb* mRNAs even when the eIF4A binding site was destroyed by mutation [5,6]. Instead, the RNA-binding activity of Pdcd4 was required to suppress translation of these RNAs, suggesting that direct RNA-binding by Pdcd4 plays a key role. Additional work revealed that the nucleotide sequences responsible for Pdcd4-induced translation suppression were located in the coding regions of c-*myb* and A-*myb* mRNAs. Furthermore, *in vitro* RNA-binding studies demonstrated that the “Pdcd4 response regions” of the c-*myb* and A-*myb* mRNAs are able to form secondary structures which were preferentially bound by Pdcd4. Overall, these experiments suggested that Pdcd4 is able to suppress translation by a novel mechanism, which involves direct binding of Pdcd4 to specific target RNAs.

How does Pdcd4 suppress translation from within the coding region? When the c-*myb* coding region was placed under the control of the Hepatitis C virus internal ribosomal entry site (HCV-IRES) Pdcd4 failed to suppress translation [5]. Because the HCV-IRES does not depend on the translation initiation factors required for cap-dependent initiation, this suggested that Pdcd4 suppresses translation of c-*myb* mRNA by interfering with one of these factors (except eIF4A) at translation initiation. However, when the c-*myb* and A-*myb* “Pdcd4 response regions” were fused to GFP RNA to ask if they are able to convey Pdcd4-responsiveness onto a heterologous RNA, a surprising observation was made. It was indeed found that Pdcd4 was able to suppress translation of the recombinant RNA, but only when a continuous open reading frame extended from the GFP coding sequence into the added c-*myb* or A-*myb* sequences. In other words, Pdcd4 suppressed the translation of the recombinant RNAs only when the binding region for Pdcd4 was itself part of the open reading frame. This observation was confirmed by introducing in-frame translational stop codons into the authentic c-*myb* and A-*myb* coding regions upstream of the “Pdcd4 response regions”. This completely abolished the inhibitory effect of Pdcd4, again indicating that Pdcd4 supresses translation only when the sequence to which it binds is part of the translated region [6]. Thus, truncating the coding region by a single stop codon is sufficient to abrogate Pdcd4-dependent inhibition. A straightforward explanation for this observation is that Pdcd4 suppresses translation of these RNAs at the elongation step (Figure 1b).

How can this be reconciled with the fact that the translation of the c-*myb* coding region was not suppressed by Pdcd4 when translation was initiated at the HCV-IRES? A possible explanation comes from the observation that the efficiency of IRES-dependent translation initiation was much lower than cap-dependent translation [5]. If the inhibition of translation elongation by Pdcd4 is augmented with increasing translation efficiency, it suggests that Pdcd4 acts like a self-adjusting controller, that limits the translation output when the translation rate is high but has no effect when it is low.

How Pdcd4 actually suppresses translation at elongation is currently unknown. Binding of Pdcd4 could stabilize RNA secondary structures and thereby hinder the passage of approaching ribosomes. Pdcd4 also interacts with the poly(A) binding protein which, in turn, could stabilize the binding of Pdcd4 to the response region [7]. Elongating ribosomes might also be blocked in an active manner. Such a mechanism has been described for the cytoplasmic polyadenylation element binding protein CPEB2, which interacts with the elongation factor eEF2 and reduces eEF2/ribosome-triggered GTP hydrolysis, thereby slowing down translation elongation of CPEB2-bound RNAs [8]. In any case, the work discussed here has led to a new paradigm for translational suppression and recognition of specific target RNAs by Pdcd4. Exploring its relevance to the function of Pdcd4 as a tumor suppressor will now be an important task.
